# Polychlorinated Biphenyls 105 and 118 Form Thyroid Hormone Receptor Agonists after Cytochrome P4501A1 Activation in Rat Pituitary GH3 Cells

**DOI:** 10.1289/ehp.10328

**Published:** 2007-08-16

**Authors:** Kelly J. Gauger, Stefanie Giera, David S. Sharlin, Ruby Bansal, Eric Iannacone, R. Thomas Zoeller

**Affiliations:** 1 Molecular and Cellular Biology Program, University of Massachusetts Amherst, Massachusetts, USA; 2 Pioneer Valley Life Science Institute, Baystate Medical Center, Spingfield, Massachusetts, USA; 3 Institute of Pharmacology and Toxicology, Department of Toxicology, University of Tübingen, Tübingen, Germany; 4 Department of Biology, Morrill Science Center, University of Massachusetts, Amherst, Massachusetts, USA; 5 Fairleigh Dickinson University, Madison, New Jersey, USA

**Keywords:** AhR, CYP1A1, endocrine disruption, PCB metabolism, thyroid hormone

## Abstract

**Background:**

Polychlorinated biphenyls (PCBs) may interfere with thyroid hormone (TH) signaling by reducing TH levels in blood, by exerting direct effects on TH receptors (TRs), or both.

**Objective:**

Our objective was to identify individual PCBs that directly affect TH signaling by acting on the TR.

**Methods:**

We administered a mixture of six PCB congeners based on their *ortho* substitution pattern, including PCBs 77 and 126 (non-*ortho*), PCBs 105 and 118 (mono-*ortho*), and PCBs 138 and 153 (di-*ortho*), to pregnant Sprague-Dawley rats from gestational days (G) 6 to 16. This mixture, or various combinations of the components, was also evaluated in a transient transfection system using GH3 cells.

**Results:**

The mixture reduced serum TH levels in pregnant rats on G16 but simultaneously up-regulated the expression of malic enzyme in liver. It also functioned as a TR agonist *in vitro*; however, none of the individual PCB congeners comprising this mixture were active in this system. Using the aryl hydrocarbon receptor (AhR) antagonist α-naphthoflavone, and the cytochrome P450 (CYP)1A1 antagonist ellipticine, we show that the effect of the mixture on the thyroid hormone response element required AhR and CYP1A1.

**Conclusions:**

We propose that PCB 126 induces CYP1A1 through the AhR in GH3 cells, and that CYP1A1 activates PCB 105 and/or 118 to a form a compound that acts as a TR agonist. These data suggest that some tissues may be especially vulnerable to PCBs interfering directly with TH signaling due to their capacity to express CYP1A1 in response to coplanar PCBs (or other dioxin-like molecules) if sufficient mono-*ortho* PCBs are present.

Polychlorinated biphenyls (PCBs) are a class of industrial compounds consisting of paired phenyl rings with various degrees of chlorination (Chana et al. 2002). Although their production was banned in the 1970s after more than a billion kilograms of PCBs were produced (Erickson 2001), they remain ubiquitous, persistent environmental contaminants that are routinely found in samples of human and animal tissues (Fisher 1999). In addition, biomonitoring studies continue to indicate that PCB levels in maternal and cord blood remain significant (e.g., Chen et al. 2006; De Saeger et al. 2005; DeCaprio et al. 2005; Furst 2006; Otake et al. 2007). Several epidemiological studies have reported an association between PCB body burden and neurodevelopmental deficits in neonates, infants, and school children (Grandjean et al. 2001; Jacobson and Jacobson 1996; Jacobson et al. 1990; Koopman-Esseboom et al. 1996; Rogan and Gladen 1992; Rogan et al. 1986). PCBs may exert a number of actions on the developing nervous system (Schantz et al. 2003). One potential mechanism by which PCBs may produce neurotoxic effects is by interfering with the ability of thyroid hormone (TH) to direct normal development.

TH is essential for normal brain development both before and after birth. Studies focused on the effects of TH insufficiency in neonates and infants indicate that the effects depend both on the severity and on the timing of low TH (Zoeller and Rovet 2004). Thus, if PCBs interfere with TH signaling to such an extent that development is compromised, the neurological or cognitive domains affected will likely reflect the timing and amount of PCB exposure. However, the specific effects of PCBs on neurological or cognitive domains potentially will also depend on the mechanism(s) by which PCBs interfere with TH signaling.

PCBs may interfere with TH signaling solely by causing a state of relative TH insufficiency. In animal studies, PCB mixtures or individual congeners can significantly reduce circulating total (Bastomsky 1974, 1976; Brouwer et al. 1998; Ness et al. 1993; Seo et al. 1995) and free thyroxine (T_4_; e.g., (Hallgren and Darnerud 2002; Morse et al. 1996), as well as serum triiodothyronine (T_3_) (e.g., Roegge et al. 2006). Some studies report that serum thyroid-stimulating hormone (TSH) is elevated by PCBs in response to low T_4_ (Fisher et al. 2006), whereas others report essentially no effect of PCB exposure on serum TSH (Hood et al. 1999). PCB exposure may also impact thyroid status in humans. Several studies have identified a negative association between PCB body burden and various measures of thyroid function (Langer et al. 2005, 2007; Persky et al. 2001; Wang et al. 2005). However, other studies have found a positive association between PCB body burden and TH levels (e.g., Otake et al. 2007), and still others find no association [see review by Hagmar (2003)]. Thus, at least part of the ability of PCBs to produce neurotoxic effects may be attributable to their ability to reduce serum thyroid hormone levels.

If PCBs act by reducing TH levels, then their effects should mimic those of TH insufficiency produced by other kinds of drugs or conditions such as propylthiouracil or low iodine. However, the effects of PCBs on developing animals are not fully consistent with effects of low TH [reviewed by Roegge et al. (2006)]. For example, although PCBs (Aroclor 1254, A1254) can reduce serum TH levels to below the limit of detection for a sensitive radioimmunoassay in rat pups, body weight was not reduced as it would have been if TH levels had been reduced with propylthiouracil (e.g., Zoeller et al. 2000). In contrast, some effects of PCBs appear as though they have a slight thyromimetic effect (Roegge et al. 2006). PCBs can increase the expression of the TH-response gene *RC3* (Gauger et al. 2004; Zoeller et al. 2000) and can produce a small but significant effect on Purkinje cell height (Roegge et al. 2006). Thus, it is possible that some individual PCB congeners, or classes of congeners, can directly interact with the TH receptor.

A significant challenge to identifying PCB congeners that may act as direct TR analogues is that there are 209 individual PCB congeners (and their metabolites), based on the pattern of chlorine substitutions, representing a potentially very large candidate pool. However, these PCB congeners can be broadly categorized according to their dioxin-like activity in that PCBs with zero or one *ortho* chlorine, two *para* chlorines, and at least two *meta* chlorines; these congeners can adopt a planar structure similar to that of dioxin (tetrachlorodibenzo-*p*-dioxin) and can bind to and activate the aryl hydrocarbon receptor (AhR) (Kodavanti and Tilson 1997; Tilson and Kodavanti 1997). In contrast, di-*ortho-*substituted PCBs may adopt a noncoplanar conformation that does not act through the AhR but nevertheless produce neurotoxic effects (Fischer et al. 1998; Seegal and Shain 1992).

Considering the complexity of PCB congener profiles in commercial mixtures, we developed a limited mixture of PCB congeners that could be studied both *in vivo* and *in vitro.* We selected six PCB congeners on the basis of their molecular structure and abundance in human tissues, representing coplanar PCBs (PCBs 77 and 126), mono-*ortho*-substituted PCBs (PCBs 105 and 118), and di-*ortho*-substituted PCBs (PCBs 138 and 153) (Figure 1). We then combined these six PCB congeners into a mixture; the relative proportion of each congener was based on their proportion in the technical mixture A1254 (Frame et al. 1996) because we have used A1254 in previous studies and this would serve as a frame of reference. We evaluated whether this mixture could exert thyroid hormone-like effects *in vivo* and in a rat pituitary cell line, GH3 cells.

## Materials and Methods

### Chemicals

**Figure 1:**
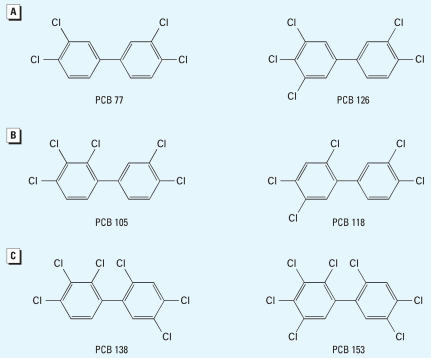
Chemical structures of PCB congeners that constitute the mixture used in the animal studies as described in the text. (*A*) Non-*ortho* PCB congeners: 3,3’,4,4’-tetrachlorobiphenyl, PCB 77, and 3,3’,4,4’,5-pentachlorobiphenyl, PCB 126. (*B*) Mono-*ortho* PCB congeners: 2,3,3’,4,4’-pentachlorobiphenyl, PCB 105, and 2,3’,4,4’,5-pentachlorobiphenyl, PCB 118. (*C*) Di-*ortho* PCB congeners: 2,2’,3,4,4’,5’-hexachlorobiphenyl, PCB 138, and 2,2’,4,4’,5,5’-hexachlorobiphenyl, PCB 153.

Individual PCB congeners (Figure 1; PCBs 77, 105, 118, 126, 138, and 153) and methanol were purchased (AccuStandard Inc., New Haven, CT, USA). The percentage of detectable impurities reported by the manufacturer were 0, 0, 0.5, 0.6, 0, and 0%, respectively. The AhR antagonist (α-naphthoflavone, α-NF), dimethylsulfoxide (DMSO), L-3,3′,5-triiodothyronine (T_3_), and the cytochrome P450 (CYP)1A1 antagonist (ellipticine) were purchased (Sigma-Aldrich Co., St Louis, MO, USA). The CYP1B1 antagonist (2,3′,4,5′-tetramethoxystilbene, TMS) was purchased (Cayman Chemical, Ann Arbor, MI, USA).

### Animals

Animals were treated humanely and with regard for alleviating suffering; all procedures were performed in accordance with the National Institutes of Health guidelines for the ethical treatment of animals and were approved by the University of Massachusetts-Amherst Institutional Animal Care and Use Committee before initiating these studies. Timed-pregnant Sprague-Dawley rats (*n* = 18; Zivic-Miller Laboratories, Inc. (Zelienople, PA, USA) arrived in our animal facility 2 days after insemination (gestational day 2, G2). The animals were individually housed in plastic cages with food and water provided continuously, and maintained on a 12-hr:12-hr light cycle (0600–1800 hours). Beginning on the day of arrival, each dam was weighed in the morning and provided with a single wafer (Keebler Golden Vanilla Wafers, Battle Creek, MI, USA) 1 hr before lights off. Beginning on G6 and continuing daily until sacrifice on G16, the dams were weighed in the morning and provided with a wafer dosed with 0.5 μL/g body weight of a solution calibrated to deliver specific doses of PCB Mix 6 (Table 1). Wafers were dosed individually each morning based on the dam’s weight. The PCB mixture was dissolved in contaminant-free methanol, pipetted onto the wafer, and allowed to dry in a fume hood throughout the day before feeding. Control wafers were dosed with methanol alone.

### Radioimmunoassay

Total T_4_ was measured in 5 μL of rat serum using a barbital buffer system. Briefly, each assay tube contained 100 μL barbital buffer [0.11 M barbital pH 8.6, 0.1% wt/vol 8-anilino-1-napthalene–sulfonic acid ammonium salt (ANS), 15% bovine γ-globulin Cohn fraction II, 0.1% gelatin], 100 μL anti-T_4_ (rabbit, Sigma-Aldrich Co.; diluted to provide a final concentration of 1:30,000), and 100 μL ^125^I-labeled T_4_ (PerkinElmer, Inc., Waltham, MA, USA). Standards were prepared from T_4_ (Sigma-Aldrich Co.) measured using a Cahn electrobalance (Cahn Instruments, Madison, WI, USA); standards were run in triplicate, whereas samples were run in duplicate. Standards were calibrated to be able to measure serum T_4_ levels from 0.4 to 25.6 μg/dL. Tubes were incubated at 37°C for 30 min, then chilled on wet ice for 30 min. Bound counts were precipitated by adding 300 μL ice-cold polyethylene glycol 8000 (20% wt/wt; Sigma-Aldrich Co.). Tubes were centrifuged at 1,800 × *g* for 20 min at 4°C, the supernatant was aspirated, and the pellet counted in a gamma counter (Packard Cobra II; PerkinElmer Inc.). The assay was run at 40–50% binding; nonspecific binding was generally below 8%. The assay was validated for rat serum by demonstrating parallelism between the standard curve and a dilution series of rat serum. The two slopes did not vary significantly as evaluated by *t*-test for two slopes (data not shown). The variability within the assay was determined by running 10 replicates of three different serum samples that represent a low, medium, and high value on the standard curve. The coefficient of variance (CV) for 0 ng/mL = 0.9%; for 3.2 μg/dL, CV = 4.7%; and for 25.6 μg/dL, CV = 3.8%. All experimental samples were evaluated in a single assay

### Transient transfection assays

GH3 cells were obtained from the American Type Tissue Collection (ATTC; Rockville, MD, USA) and were maintained in Hams F-12K media supplemented with 100 U/mL penicillin, 100 μg/mL streptomycin, 2 mM l-glutamine (Mediatech, Herndon, VA, USA), and 10% fetal bovine serum (FBS; Hyclone, South Logan, UT, USA) in a 37°C humidified incubator with 5% CO_2_.

Cells at 70% confluence were plated at a density of 2 × 10^5^ cells per well in 24-well plates 24 hr before transfections using Superfect (QIAGEN, Valencia, CA, USA) according to the manufacturer’s instructions. Cells were transfected with a DR4-tk-Luc firefly luciferase vector [kindly provided by D. Darling (Cabanillas et al. 2001)], a mutated DR4 (ΔDR4-tk-Luc firefly luciferase vector provided by A. Hollenberg, Harvard University, Cambridge, MA) plus the pRL-CMV renilla luciferase vector (Promega Corp., Madison, WI, USA) to control for transfection efficiency. The sequence of the DR4 promoter is 5′-ttatAGGTCAcatgAGGTCAagtt-3′; the ΔDR4 was different by a single base 5′-ttatAGATCAcatgAGGTCAagtt-3′ (capital letters are half-sites; note base difference in the first half-site ΔDR4). Twenty-four hours after transfection, the media was removed, washed with 1× phosphate-buffered saline (PBS), and replaced with media containing 10% AG 1-X8 resin (analytical grade; Bio-Rad Laboratories, Hercules, CA, USA) treated FBS [to remove T_3_ from the serum (Samuels et al. 1979)]. Cells were treated by replacing stripped media with media containing various compounds as described below in individual experiments. PCBs were dissolved in DMSO and T_3_ was dissolved in ethanol (EtOH); the final concentration of vehicle was always < 0.168%. The concentration of PCBs is shown in Table 4. After 24-hr incubation, the media was removed, cells were washed with 1× PBS, and lysed using passive lysis buffer (Promega Corp.). Luciferase activity was detected using the Dual-Luciferase‚ Reporter Assay System (Promega Corp.) according to the manufacture’s instructions, and the light output was measured with a luminometer (MONLIGHT 1500; Analytical Luminescence Laboratory, San Diego, CA, USA). All experiments were performed independently 3 times, with treatments performed in triplicate for each experiment.

### *Ethoxyresorufin-*O*-deethylation (EROD) assay*

The EROD assay was performed according to Peters et al. (2004), a modification of the method described by Burke and Mayer (1974). After 24 hr, the media was removed and the cells were washed twice with 1× PBS and serum-free medium containing 5 mM MgCl_2_, 5 μM 7-ethoxyresorufin (BIO-MOL International LP, Plymouth Meeting, PA, USA), and 10 μM dicumarol (Sigma-Aldrich Co.) was added to each well. The conversion of 7-ethoxyresorufin to resorufin, which has an excitation and emission wavelength of 544 nm and 590 nm, respectively, was followed fluorometrically at 37°C over a 10-min period using a POLARstar OPTIMA plate reader (BMG LABTECH GmbH, Offenburg, Germany). A standard curve relating fluorometric units to resorufin (Sigma-Aldrich Co.) concentrations was used to convert the observed fluorometric units to picomoles of resorufin formed. After the fluorometric readings for resorufin were taken, the reaction mixture was aspirated and the cells were lysed with CelLytic-M (Sigma-Aldrich Co.) to obtain protein measurements using a BCA assay kit (Pierce Biotechnology, Inc., Rockford, IL, USA).

### RNA isolation

Total RNA was extracted from the liver of dams, or from GH3 cells, using an acid–phenol extraction procedure (Chomczynski and Sacchi 1987), according to the manufacturer’s instructions (Trizol; Invitrogen Corp., Carlsbad, CA, USA), followed by standard phenol/chloroform extraction. The final RNA pellet was resuspended in 0.1% sodium dodecyl sulfate or nuclease-free water. Total RNA was quantified by ultraviolet spectrophotometry and the integrity confirmed by gel electrophoresis.

### Real-time polymerase chain reaction (PCR) assay

Relative levels of mRNA were determined by quantitative real-time PCR using the Mx3000P real-time PCR system (Stratagene, La Jolla, CA) and primer pairs/probes described in Table 2. The assay for malic enzyme (ME) expression was performed in 10 μL of 1× QuantiTect SYBR RT-PCR Master Mix (QIAGEN GmbH, Hilden, Germany) containing 200 nM forward primer, 200 nM reverse primer, and 100 ng of total RNA. The assay for CYP1A1 and CYP1B1 expression was performed in 10μL of 1× QuantiTect Probe RT-PCR Master Mix (QIAGEN GmbH) containing 400 nM forward primer, 400 nM reverse primer, 200 nM probe, and 1 μg of total RNA. The conditions for cDNA synthesis and target mRNA amplification were performed as follows: 1 cycle of 50°C for 30 min; 1 cycle of 95°C for 15 min; and 45 cycles each of 94°C for 15 sec, 58°C (CYP1A1/CYP1B1) or 60°C (ME) for 30 sec, and 76°C for 30 sec. All values were normalized to the amplification of β-actin mRNA, which was performed in parallel wells for each treatment and real-time PCR analysis was performed in duplicate wells for each treatment.

### Statistical analysis

The *in vivo* results were analyzed using a one-factor analysis of variance (ANOVA). The *in vitro* data were analyzed using a Student *t*-test or two-factor ANOVA. Post-hoc tests, where appropriate, were performed using the Bonferroni *t*-test, where the mean squared error term in the ANOVA table was used as the point estimate of the pooled variance (SuperAnova software; Abacus Concepts, Inc., Berkley, CA, USA).

## Results

### Dams

**Figure 2:**
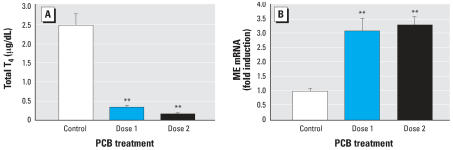
Effect of PCB treatment on serum total T_4_ (*A*) and ME mRNA in liver (*B*) of pregnant Sprague-Dawley rats at the time of sacrifice on G16. Error bars represent mean ± SE (*A*) or mean ± SE ME/β-actin (*B*). Numbers of animals in each group are as follows: (*A*) Control, 6; Dose 1, 8; Dose 2, 8. (*B*) Control, 5; Dose 1, 6; Dose 2, 7. See “Materials and Methods” for treatment details. **p < 0.01, significantly different from control group using the Bonferroni *t*-test after one-way ANOVA.

Exposure to the defined mixture of six PCBs significantly reduced circulating levels of total T_4_ in G16 dams (Figure 2A; *F*_2,31_ = 22.5, *p* = 0.0001). Serum T_4_ was reduced to a similar extent in animals exposed to both doses of the PCB Mix 6. Despite this reduction in serum total T_4_, real-time PCR analysis of RNA extracted from maternal liver revealed that ME mRNA levels were significantly increased in animals treated with both doses of the PCB Mix 6 (Figure 2B; *F*_2,18_ = 17.730, *p* = 0.001). Like the effects of this PCB mixture on serum total T_4_, there was no apparent difference in the ability of the two doses of the PCB Mix 6 to induce ME expression in the maternal liver.

### GH3 cells

The *in vivo* results indicated that one or more of the PCB congeners present in this defined mixture of six PCBs could act as a direct agonist on the TR. To test this hypothesis, we evaluated the activity of the PCB mixture and the individual components in a transient transfection system using a rat somatomamatroph cell line (GH3) transfected with a reporter gene (luciferase) driven by a canonical TH response element (TRE; direct repeat with a four-base spacer, DR4).

**Figure 3:**
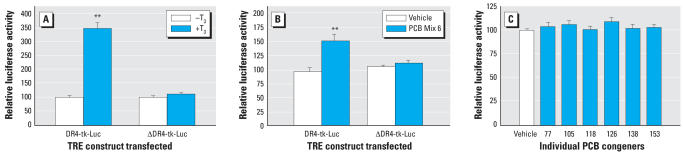
Effects of 1 × 10^−7^ M T_3_ (*A*), 10 μM PCB Mix 6 (*B*), or individual PCB congeners (see Table 4 for concentrations) (*C*) treatments on relative luciferase activity in GH3 cells. Error bars represent mean ± SE of relative luciferase activity normalized to control wells. All treatments were performed in triplicate, and the final results obtained from three separate experiments. Values are reported as percent control for the purpose of illustration. **p < 0.01, significantly different from control (vehicle treatment) group using a Student *t*-test.

To establish the response characteristics of this system, we first evaluated the effect of 1 × 10^−7^ M T_3_, and found a significant increase (3.46 ± 0.42-fold) in luciferase activity in cells transfected with the DR4-tk-Luc reporter plasmid (Figure 3A). This effect was blocked by a single base mutation in the DR4 TRE; T_3_ did not increase luciferase activity in cells transfected the ΔDR4-tk-Luc vector (Figure 3A). Similarly, the PCB Mix 6 (10^−5^ M) significantly increased (1.51 ± 0.21-fold) luciferase in cells transfected with DR4-tk-Luc but not in cells transfected with ΔDR4-tk-Luc (Figure 3B). These findings provide strong support for the hypothesis that one or more of the PCB congeners in this defined mixture can act as a direct agonist on the TR. However, none of the individual PCB congeners present in the defined mixture caused an increase in relative luciferase activity (Figure 3C).

Because the mixture of six PCB congeners acted as a TH agonist in GH3 cells in combination but not as individual congeners, we considered the possibility that one or more of these parent PCB congeners must be “activated” by metabolism to form TH agonists. Previous studies have shown that pituitary cells exhibit a robust cytochrome P450 response to dioxin (Huang et al. 2002, 2003); these enzymes are known to hydroxylate PCBs (Sjodin et al. 1998). Thus, it was possible that one or more hydroxylated metabolites accounted for the agonist effect of the mixture of six PCBs.

To test this hypothesis, we first characterized the response characteristics of GH3 cells to AhR agonists. Cells treated with the AhR agonist β-NF induced the expression of both CYP1A1 and CYP1B1 mRNAs in GH3 cells (data not shown) but CYP1A2 is not induced. Sequence analysis of the amplified products confirmed the authenticity of these PCR products.

**Figure 4:**
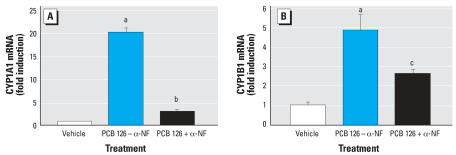
Role of AhR in PCB 126-induction of cytochrome P450 genes. Cells were treated with 10μM PCB 126 in the presence or absence of 1 x 10^−6^ M of the AhR antagonist, α-NF. The levels of CYP1A1 (*A*) and CYP1B1 (*B*) mRNAs were measured by real-time PCR. PCB 126 significantly increased CYP1A1 (*A*) and CYP1B1 (*B*) mRNAs and α-NF abrogated these effects. Error bars represent mean ± SE CYP1A1/β-actin (*A*) or CYP1B1/β-actin (*B*) mRNAs and are expressed as fold induction over vehicle alone (DMSO). ^***a***^*p* < 0.01, significantly different from control group using the Bonferroni *t*-test after one-way ANOVA. ^***b***^*p* < 0.01, ^***c***^*p* < 0.05, significantly different from PCB 126 –treated group using the Bonferroni *t*-test after one-way ANOVA.

If our defined mixture of six PCB congeners induces the expression of CYP genes that metabolize parent PCB congeners, then the dioxin-like PCBs (e.g., PCB 126) should induce a CYP response that requires the AhR. Real-time PCR analysis revealed that PCB 126 significantly increased the expression of both *CYP1A1* (20-fold, Figure 4A) and *CYP1B1* (5-fold, Figure 4B) (*F*_2,6_ = 375.656, *p* = 0.0001; *F*_2,6_ = 17.585, *p* = 0.0031, respectively), and that α-NF significantly blocked the effect of PCB 126 (Figure 4) on the induction of both genes.

Considering that PCB 126 could induce the expression of CYP enzymes that could metabolize PCBs, we next tested whether AhR activation and induction of these P450 enzymes were required for TH agonist activity in GH3 cells. To test this hypothesis, we used ellipticine or TMS to block CYP1A1 or CYP1B1 activity, respectively. To confirm that these drugs would block the expected activities in GH3 cells, we first evaluated the effect of PCB 126 on EROD activity (Table 3). GH3 cells were treated with PCB 126 (10^−5^ M) in the presence or absence of ellipticine or TMS (1 × 10^−7^ M to 1 × 10^−5^ M). PCB 126 significantly increased EROD activity in GH3 cells, and this was completely blocked by the addition of either ellipticine or TMS. All doses of ellipticine (10^−7^–10^−6^ M) completely blocked PCB 126–induced EROD activity; thus, we used the lowest dose in the following experiments. In contrast, the lowest dose of TMS (10^−7^ M) did not completely block the PCB 126–induced EROD activity in GH3 cells; therefore, we used 10^−6^ M TMS in the following experiment.

**Figure 5:**
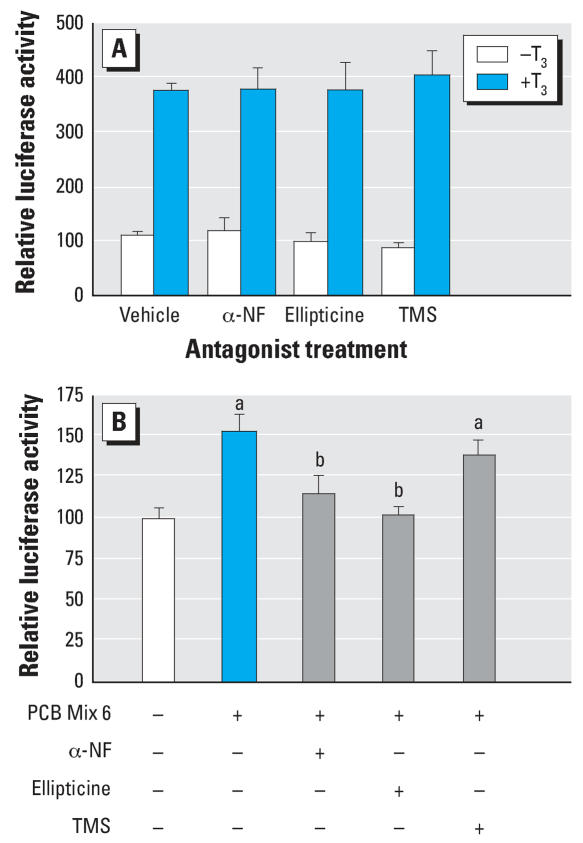
Effects of cytochrome P450 antagonsits on T_3_-induced (*A*) and PCB Mix 6–induced (*B*) relative luciferase activity in GH3 cells. “–” indicates no treatment; “+” indicates treatment with compound shown left of row. α-NF and TMS were used at concentrations of 10^−6^ M; ellipticine was used at 10^−7^ M. Error bars represent mean ± SE of relative luciferase activity reported as percent control for the purpose of illustration. ^***a***^*p* < 0.01, significantly different from control group using the Bonferroni *t*-test after one-way ANOVA. ^***b***^*p* < 0.01, significantly different from PCB Mix 6–treated group using the Bonferroni *t*-test after one-way ANOVA.

These experiments showed that PCB 126 could induce AhR-dependent CYP1A1, and to a much lesser extent CYP1B1 expression in GH3 cells, and that these effects could be blocked by α-NF, ellipticine or TMS. To test whether the ability of the PCB Mix 6 to activate luciferase activity through the canonical TRE required AhR-induced CYP activity, we employed these drugs to block AhR, CYP1A1, or CYP1B1. However, in principle, these drugs may also interfere with the TR; thus, we first demonstrated that α-NF, ellipticine, or TMS, did not interfere with the ability of T_3_ to induce luciferase activity in GH3 cells (Figure 5A). We found that the PCB Mix 6 (10^−5^ M) significantly increased luciferase activity in GH3 cells (*F*_(2,15)_ = 7.229, *p* = 0.0002), and this was significantly reduced by concurrent treatment with 1 × 10^−6^ M α-NF or 1 × 10^−7^ M ellipticine. In contrast, concurrent treatment with 1 × 10^−6^ M TMS did not significantly inhibit the effect of PCB 126 (Figure 5B).

**Figure 6:**
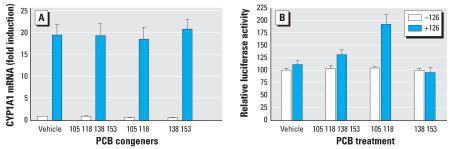
Effects of PCB congener combinations on expression of CYP1A1 mRNA (*A*) and TR-mediated relative luciferase activity (*B*) in GH3 cells. (*A*) PCB concentrations are described in Table 4; CYP1A1 mRNA was measured by real-time PCR. Only treatment groups that included PCB 126 significantly increased CYP1A1 expression. (*B*) PCB congener concentrations are described in Table 4. Error bars represent mean ± SE of relative luciferase activity and values are reported as percent control for the purpose of illustration. An increase in relative luciferase activity was observed when cells were PCBs 126, 105, and 118. **p < 0.01 [significantly different from corresponding PCB combination group not treated with PCB 126 (*A*) or group treated with PCBs 118 and 105 (*B*) using the Bonferroni t-test after two-way ANOVA].

These findings support the hypothesis that the ability of this defined mixture of six PCBs to activate the TR depends on AhR-induced CYP1A1. Because PCB 126 is known to bind to and activate the AhR (Toyoshiba et al. 2004), we first tested whether PCB 126 was the most potent AhR-agonist in the mixture. Real-time PCR analysis revealed that the expression of CYP1A1 was up-regulated only when PCB combinations included PCB 126 [*F*_1,20_ = 0.0195, *p* = 0.0001 (Figure 6A)]. These findings indicated that, in the Mix 6, PCB 126 was the dominant inducer of CYP1A1. To test whether PCB 126 is required to be present in a minimal mixture of PCBs to activate the DR4-tk-LUC construct in GH3 cells, we tested the same PCB combinations for their ability to drive luciferase activity from the DR4. Interestingly, relative luciferase activity was increased only in GH3 cells treated with the combination of PCBs 126, 105, and 118 (Figure 6B; *F*_1,67_ = 4.371, *p* = 0.0404). Post-hoc analysis using the Bonferroni *t*-test revealed that cells treated with PCBs 126, 105, and 118 exhibited a significantly higher relative luciferase signal than cells treated with PCBs 105 and 118 alone.

## Discussion

Previous studies have reported that PCB mixtures can have paradoxical effects on TH signaling, indicating that at least some PCB congeners or their metabolites can exert a direct action on the TH receptor (Zoeller 2005). We now show that a limited mixture of only six PCB congeners can reduce serum TH levels at the same time that it increases the expression of a well-known TH-response gene, ME, in the liver; thus, this limited PCB mixture can recapitulate the effect of a complex technical mixture, A1254 (Gauger et al. 2004; Zoeller et al. 2000). In addition, this limited mixture exerted a TH-like action *in vitro* only when present as a mixture and not when present as individual parent congeners. This unexpected result was found to be due to a requirement for AhR activation and CYP1A1 expression and activity, which is not uniformly induced by the various individual congeners. Thus, we propose a two-step process in which an AhR ligand (e.g., PCB 126) induces CYP1A1, which then acts on noncoplanar PCBs (e.g., PCBs 105 and 118), producing analogues that activate the TR. This mechanism may account for tissue- or cell-specific differences in the effect of PCB exposure on TH signaling.

These data show that PCBs can exert a TR agonist effect both *in vivo* and *in vitro*. This interpretation *in vivo* is based on the observation that this mixture increased the expression of ME mRNA in the liver. The *ME* gene is well known to be a direct target of thyroid hormone action (e.g., Yin et al. 2005). Thus, we reasoned that one or more of the six PCBs that made up our mixture would exert an agonist effect on the TR in GH3 cells. This PCB mixture was designed to include two each of the non-*ortho*, mono-*ortho*, and di-*ortho* substituted PCBs. In addition we assembled the mixture using the relative proportions defined for A1254 by Frame et al. (1996), and a mass of the total mixture that would be contained in a dose of 8 mg/kg A1254 that we have previously shown to be effective in producing TH-like effects (Zoeller et al. 2000). Because we also considered the possibility that the mass of PCBs given was important, our second dose was set to 4 mg, with the individual congeners being assembled in the same relative proportion of dose 1. Interestingly, we did not find a dose–response effect on serum T_4_, which likely indicates that dose 1 was sufficient to reduce serum total T_4_ to a very low level that was not further reduced by doubling the dose of the mixture.

Our studies in GH3 cells demonstrate that the defined PCB mixture can exert an agonist effect on the rat TR. GH3 cells are well known to be sensitive to TH (e.g., Ghisari and Bonefeld-Jorgensen 2005), and they express both TRαand TRβ (confirmed in our studies by PCR; data not shown). In addition, the luciferase construct we employed was based on the canonical TH response element, a DR4, which binds to the TR to drive gene expression (Quack et al. 2002). Finally, we confirmed the specificity of this TRE using the ΔDR4 construct that does not bind to the TR or mediate TH-dependent gene expression. Considering this, it was surprising to find that the PCB mixture could exert a TH agonist effect in GH3 cells, but that none of the PCB congeners could exert such an action when tested alone.

There were at least two explanations for this finding. First, because we used a concentration of individual PCB congeners present as a component in the full mixture (Table 4), it was possible that the dose of each congener was additive on the TR in the mixture, but the concentration of individual congeners was not sufficient to produce an effect alone. In contrast, it was possible that different PCB congeners have different effects, with coplanar PCBs capable of acting on the AhR and inducing the expression of metabolic machinery that could modify other PCB congeners in the mixture to be able to act on the TR in the concentrations present in the mixture. We reasoned that we could discriminate between these hypotheses using a series of experiments that tested whether AhR activation and CYP expression are required for TR agonist activity and that used various combinations of mono-and di-*ortho* substituted congeners.

First, we verified that GH3 cells express CYP1A1 and CYP1B1 in response to a known AhR agonist, β-NF. In addition, we showed that PCB 126 induces CYP1A1 and CYP1B1, and that this was associated with an increase in EROD. Finally, we also verified that these effects of PCB 126 were blocked by ellipticine, TMS, or by the AhR antagonist α-NF. Therefore, the finding that the AhR antagonist α-NF and the CYP1A1 antagonist ellipticine blocked the ability of the mixture of six PCB congeners to activate DR4-tk-Luc demonstrated that AhR activation and CYP1A1 expression was necessary for this mixture to act on the TR. However, these drugs did not alter the ability of T_3_ to activate the DR4-tk-Luc. Considering this, we reasoned that PCB 126 was necessary, but not sufficient, for TR activation. This interpretation was confirmed by showing that the mixtures of all noncoplanar PCBs together (i.e., PCBs 105, 118, 138, and 153), or separated by their *ortho*-substitution pattern (i.e., PCBs 105/118 or 138/153), required the presence of PCB 126 to activate the DR4 construct in GH3 cells. These observations strongly support the hypothesis that PCB 126 induces the expression of metabolic enzymes that “activate” noncoplanar PCBs to form TR agonists in GH3 cells.

Interestingly, only the combination of PCBs 105 and 118 contained the full TH-like effect of the mixture of six PCB congeners when combined with PCB 126. This is perplexing because this effect was not observed when these congeners were combined with PCBs 138 and 153. This mixture of five PCB congeners was only missing PCB 77 from the original mixture of six PCBs; thus, it is not immediately obvious why the mixture that did not contain PCB 77 would not exhibit TH-like activity.

PCBs 105 and 118 are metabolized to form 4-hydroxy-2,3,3′,4′,5-pentachlorobiphenyl (4-OH-PCB107) (Sjodin 1998); thus, the current data indicate that 4-OH-PCB107 may be an important TR agonist. This particular PCB metabolite also is abundant in PCB-exposed humans and cord blood, rats and their fetuses, Baltic seals, and white-tailed eagles (Bergman et al. 1994; Letcher et al. 1999; Meerts et al. 2002; Sandau et al. 2002; Sjodin 1998; Sjodin et al. 2000). In fact, 4-OH-PCB107 metabolite levels are higher in children than in their mothers (Fangstrom et al. 2005). Several studies have found that hydroxylated PCB metabolites can affect the TH receptor. We have shown that 4-OH-PCB106 can act as a direct TR agonist in GH3 cells (You et al. 2006). Kitamura et al. (2005) reported that nine separate hydroxylated PCB congeners can bind to the rat TR with an IC_50_ (half-maximal concentration) as low as 5 μM. These hydroxylated PCBs included those with low (trichloro) to high (septa-chloro) chlorine substitution patterns. Arulmozhiraja et al. (2005) identified several PCB congeners that exhibit weak TH activity in a yeast two-hybrid assay optimized to identify such activity. Thus, PCB hydroxylation *in situ* may be an important mechanism by which PCBs can interfere with TH action in tissues.

Not all investigators report that PCBs act as agonists on the TR. Kimura-Kuroda et al. (2005, 2007) found that several hydroxylated PCBs interfere with T_3_-dependent neurite outgrowth in mouse cerebellar Purkinje cell primary cultures. In addition, Bogazzi et al. (2003) found that a commercial mixture of PCBs (A1254) inhibited TR action on the ME promoter in a chloramphenicol acetyl-transferase assay. Similarly, Iwasaki et al. (2002) found that a specific hydroxylated PCB congener inhibits TR-mediated transcriptional activation in a luciferase assay at concentrations as low as 10^−10^ M. These findings do not necessarily conflict with findings that PCBs can act as TR agonists. As imperfect TH analogues, PCBs may well exert different actions on the TR on different DNA regulatory elements or in different cell types. Therefore, taken together, these findings indicate that a wide array of hydroxylated PCB metabolites may exert direct actions on the TR, and their production in specific cell types by the process we have identified may be an important element in the toxicity of PCBs.

In conclusion, the data presented here indicate that specific PCBs are metabolized in rat pituitary GH3 cells to form TR agonists. The metabolic machinery responsible for this metabolism is induced by PCBs that are not themselves metabolized to form TR agonists. These data suggest that different cell types and tissues may respond differently to PCB exposure, depending on their ability to express these P450 proteins. In addition, these findings suggest that PCB metabolites may become sequestered in cells that perform these metabolic steps; that is, PCBs may gain entry into cells by a mechanism that is no longer available to them for exit when they have been modified. Although speculative, it would help explain why different tissues are differentially contaminated with specific PCB metabolites. Finally, these data also indicate that epidemiological studies should evaluate the association of thyroid hormone end points with the combination of exposures to TEQ (from any contaminant source) and specific PCB congeners rather than single congeners alone.

## Figures and Tables

**Table 1:** Composition of PCB mixtures.

		PCB					
PCB treatment	Composition	77	105	118	126	138	153
Mix 6	Percent in A1254^a^	0.200	7.400	13.600	0.020	6.000	3.500
Dose 1	Congener dose (mg/kg)^b^	0.016	0.592	1.056	0.001	0.480	0.304
Dose 2	Congener dose (mg/kg)^c^	0.026	0.967	1.722	0.003	0.784	0.496

a Each value represents the percentage of A1254 (by mass) contributed by the PCB congener labeled at the top of the column, as described by Frame et al. (1996). Thus, this mixture of six PCBs represents 30.72% of the total mass of A1254.

b Each value represents the milligrams per kilogram of PCB congener delivered to each animal daily. This mixture was calibrated to deliver 30.72% of 8 mg/kg/day. Thus, the total dose of PCBs delivered to the animals was 2.46 mg/kg/day.

c Each value represents the milligrams per kilogram of PCB congener delivered to each animal daily. This mixture was calibrated to deliver a total of 4 mg/kg/day.

**Table 2:** Sequences of the CYP primer/probe sets used for PCR and quantitative PCR.

Gene	Primer	5′ →3′ sequence	Amplicon size (bp)
*CYP1A1*	Forward Reverse	CCATGACCAGGAACTATGGG TCTGGTGAGCATCCAGGACA	340
*CYP1A2*	Forward Reverse	TGCAGAAAACAGTCCAGGA GGAAAAGGAACAAGGGTGGC	794
*CYP1B1*	Forward Reverse	TGACAGACAGAGAGTGCATGAGCA TGGGTCTGGTTGGCTTAATGAGGA	495
*Malic enzyme*	Forward Reverse	AGGCCTCTTTATCAGTATCCAC CCATCCCGTACAACCAA	140
*CYP1A1*	Forward Reverse Probe	GAAGAAGCTAATCAAAGAGCACTACAGG CAATGCTCAATGAGGCTGTCTG FAM-CATTTGAGAAGGGCCACATCCGGG-BHQ	80
*CYP1B1*	Forward Reverse Probe	TGGCTGCTCATCCTCTTCACC CCCACAACCTGGTCCAACTC FAM-ATGTGCAGGCCCGAGTGCA-BHQ	73
β-*Actin*	Forward Reverse Probe	TGAACCCTAAGGCCAACCGTGAAA ATACAGGGACAACACAGCCTGGAT FAM-ATCATGTTTGAGACCTTCAACACC-BHQ	101

**Table 3:** Effect of CYP inhibitors on EROD activity induced by PCB 126 in GH3 cells.

P450 agonist (M)	P450 antagonist (M)	EROD (pmol/min/mg,mean ± SE)
—	—	8.598 ± 1.273
PCB 126 (10^−5^)	—	79.841 ± 4.243**
PCB 126 (10^−5^)	Ellipticine^a^ (10^−7^)	13.048 ± 0.182
PCB 126 (10^−5^)	Ellipticine^a^ (10^−6^)	15.442 ± 2.679
PCB 126 (10^−5^)	Ellipticine^a^ (10^−5^)	12.213 ± 0.682
PCB 126 (10^−5^)	TMS^b^ (10^−7^)	56.745 ± 6.155**
PCB 126 (10^−5^)	TMS^b^ (10^−6^)	19.867 ± 0.628
PCB 126 (10^−5^)	TMS^b^ (10^−5^)	18.273 ± 2.166

a CYP1A1 antagonist.

b CYP1B1 antagonist.

** Treatment group significantly different from control group (*p* < 0.001).

**Table 4:** Composition of PCB mixtures.

	Concentrations of PCB congeners (μM)				
PCB combinations	105	118	126	138	153
Vehicle	0.00	0.00	0.00	0.00	0.00
105 + 118 + 138 + 153	2.42	4.31	0.00	1.96	1.24
105 + 118	2.42	4.31	0.00	0.00	0.00
138 + 153	0.00	0.00	0.00	1.96	1.24
126	0.00	0.00	0.01	0.00	0.00
126 + 105 + 118 + 138 + 153	2.42	4.31	0.01	1.96	1.24
126 + 105 + 118	2.42	4.31	0.01	0.00	0.00
126 + 138 + 153	0.00	0.00	0.01	1.96	1.24

Each value represents the molar concentration of the PCB congener used for *in vitro* experiments. This mixture was calibrated to deliver a total of 10 μM PCB. In experiments in which single congeners were tested, the concentration is as shown in this table except where otherwise noted.
